# Implementing Digital Mental Health Tools in the Public Behavioral Health Systems: A Multi-City/County Evaluation of California’s Help@Hand Project

**DOI:** 10.21203/rs.3.rs-7794276/v1

**Published:** 2025-11-04

**Authors:** William Bevens, Sarah Elizabeth Stoeckl, Elizabeth Eikey, Margaret Schneider, Nicole A. Stadnick, Kai Zheng, Dana B. Mukamel, Dara H. Sorkin, Stephen M. Schueller

**Affiliations:** University of California San Diego; UC Irvine: University of California Irvine; UC San Diego: University of California San Diego; UC Irvine: University of California Irvine; UC San Diego: University of California San Diego; UC Irvine: University of California Irvine; UC Irvine: University of California Irvine; UC Irvine: University of California Irvine; UC Irvine: University of California Irvine

**Keywords:** digital mental health, implementation science, public-private partnerships, mixed-methods evaluation, equity, infrastructure

## Abstract

**Background:**

Digital mental health interventions (DMHIs) can expand access to mental health care, particularly in underserved regions. Despite this, real-world implementation is complex and few studies have investigated the factors that shape it. We provide insights from Help@Hand, a five-year implementation project deploying DMHIs within public systems across counties and cities (i.e., sites) in California. This study examined how diverse local systems implemented DMHIs within a multi-site initiative and identified factors that facilitated or prevented success.

**Methods:**

Data were collected from project leads at participating sites (N = 12) through four waves of surveys and two rounds of interviews. Surveys and interviews were informed by the Consolidated Framework for Implementation Research (CFIR) and captured implementation experiences over time, providing contextual insight into local decision-making, resource use, and coordination efforts. Interviews and surveys were analyzed using a sequential mixed-methods design using the CFIR. Reflexive thematic analysis and descriptive survey statistics were used to examine implementation strategies, challenges, and adaptations across sites.

**Results:**

Eight themes were identified: (1) vendors supplementation was a facilitator, though customization and data access often posed as barriers; (2) sustaining a workforce proved challenging due to turnover and understaffing; (3) sites operated in tight and uncertain funding environments; (4) community outreach required multimodal strategies, with in-person engagement more effective than traditional marketing; (5) new media proved essential for marketing, but was resource-intensive; (6) the project management entity provided contracting support, but introduced bureaucratic delays; (7) collaborations were both facilitated and disrupted by organizational factors; and (8) achieving digital equity is challenging, requiring considerations device access, internet connectivity, and digital literacy.

**Conclusions:**

This study provides insights into the processes underpinning the implementation of DMHIs within public behavioral health systems. Successful implementation did not merely involve providing the community with access to digital tools; it required extensive coordination within and across sites, while ensuring that technologies could be adapted to meet local needs. Future large-scale digital mental health initiatives should aim to operationalize insights from this evaluation to ensure successful and sustainable DMHIs, with structured opportunities for collaboration and shared learning to enhance organizational capacity and alignment across levels.

## Background

Digital mental health interventions (DMHIs) are increasingly recognized for their potential to expand access to care, particularly in under-resourced or geographically isolated communities. Delivered through websites, mobile apps, text messaging, and wearable technology, DMHIs have demonstrated efficacy across a range of mental health conditions (1–5) and are often promoted as scalable solutions to workforce shortages, access gaps, and behavioral health system strain (5, 6). Despite DMHIs demonstrated promise within clinical trial settings, successful translation into real-world settings remains elusive. Challenges in designing, integrating, and sustaining DMHIs, such as limited digital infrastructure, unclear reimbursement models, inadequate workforce capacity, and organizational misalignment, create significant barriers to successful implementation, especially in the United States public sector where technical resources can vary widely (7–10).

Despite these barriers, several DMHI implementations have achieved some success within the United States, such as Reliant Medical Group’s Precision Behavioral (PBH) model (11) and the upscaling of Cope Notes in Florida (12). Evidence for DMHI implementation within behavioral health systems points to several recurring obstacles. Clinicians may be hesitant to adopt new technologies without clear evidence of prior success in similar settings (13). Similarly, agencies often lack guidance for assessing fit between digital tools and their populations, particularly in low-resource settings (8) and may have legal concerns around privacy and confidentiality (14). Furthermore, access to devices, internet connectivity, and digital literacy can limit the ability for populations to access DMHIs, especially among marginalized groups (15). As a result, many implementation efforts languish in early stages resulting in small-scale pilots or research-driven deployments that do not transition to sustainable system change (16, 17).

In contrast, national health systems in other countries have demonstrated more successful examples of DMHI implementation. Australia’s MindSpot Clinic (18, 19), the UK’s “Talking Therapies” program (20), and the European Union’s ImpleMentAll initiative (21) offer examples of coordinated, system-level DMHI implementation. These initiatives underscore several facilitators of successful implementation, including centralized coordination, alignment with existing care pathways, sustained funding, and investment in workforce development. They also emphasize continuous evaluation and learning, with routine data collection guiding adaptation and accountability. Implementation research from MindSpot, IAPT, and ImpleMentAll supports the values of strong governance structures, national procurement standards, and cross-site learning in enabling successful scaling (22, 23).

California’s Help@Hand project offers a valuable opportunity to explore DMHI implementation in practice within the United States. Funded at over $80 million through the Mental Health Services Act and administered through the California Mental Health Services Authority (CalMHSA), Help@Hand represented an unprecedented statewide investment in digital mental health. The five-year project engaged 14 county and city sites, serving nearly half of California’s 39 million residents, Sites were diverse in size, geography, and resources, ranging from urban to rural regions across the entire state. Despite this variation, Help@Hand promoted common mental health learning objectives: detecting mental health needs earlier, reducing stigma, increasing access to appropriate care, promoting social connectedness, and improving data-driven service planning. Together, these objectives positioned Help@Hand as a large-scale test of digital mental health integration in a diverse public behavioral health system.

This longitudinal implementation evaluation study evaluated the barriers to and facilitators of implementation of Help@Hand initiatives within and between sites. Using a mixed-methods approach, this study analyzed four waves of survey data and two rounds of qualitative interviews conducted over five years with site project leads to identify both site-specific and shared barriers and facilitators to implementing DMHIs across disparate, independent public behavioral systems.

## Methods

### Overview

The present analysis is a sequential mixed-methods design of implementation aspects across the Help@Hand sites. Participants included project leads, those individuals directly responsible for overseeing the planning, coordination, or management of Help@Hand activities within their sites. Data collection occurred between April 2022 and November 2023 and included two rounds of semi-structured interviews and four rounds of surveys. Participating sites in the Help@Hand project included City of Berkeley, Kern County, Los Angeles County, Marin County, Modoc County, Mono County, Monterey County, Orange County, Riverside County, San Francisco County, San Mateo County, Santa Barbara County, City of Tehama, and the Tri-Cities (Claremont, La Verne, and Pomona). Several sites completed their participation in Help@Hand prior to the period of these surveys. See details in Table 1.

### Ethics, Consent, and Compensation

The evaluation protocol was reviewed and approved by the University of California, Irvine Institutional Review Board. Components were designated as exempt under the “Quality Improvement Project” classification. Verbal consent, including consent to record interviews, was obtained at the beginning of each session.

### Instrument Design and Adaptation

Instrument development was guided by the Consolidated Framework for Implementation Research (CFIR) and refined iteratively. The evaluation team used the CFIR Interview Guide Creator to develop the initial semi-structured interview guide, which was revised based on feedback from project collaborators and site implementation leads (24). Surveys assessed implementation experiences, including successes, challenges, accomplishments, learnings, and recommendations. Survey items were developed and iterated upon using interview findings, with updates reflecting emerging project activities.

### Instrument Administration

Semi-structured interviews were conducted via Zoom and typically lasted between 30 and 45 minutes. One team member led the interview while another took notes. All interviews were recorded, automatically transcribed through Zoom, and reviewed for accuracy. After each interview, the team compiled summary notes capturing initial insights and salient themes for ongoing analysis. Surveys were distributed electronically via REDCap, with follow-up reminders sent to maximize response rates. Response options included binary (e.g., “yes”/”no”) responses for successes, challenges, and accomplishments and Likert-type scales for the learnings and recommendations (e.g., “meaningful”, “very meaningful”, “moderately important”).

### Data Analysis

We employed a sequential mixed-methods design, analyzing qualitative and quantitative data independently before integrating findings in the final stage. Qualitative data was used first throughout the project to inform subsequent surveys, but at the data analytic phase quantitative analysis was completed first. This design enabled each method to contribute unique insights while supporting a comprehensive understanding.

The qualitative analysis team comprised researchers with backgrounds in clinical psychology and implementation science. The team’s experience with digital mental health research informed the interpretation of data. Reflexive discussions were held throughout coding to examine how disciplinary perspectives and prior involvement in related projects could influence theme development and interpretation.

### Interview data

Qualitative data were analyzed using reflexive thematic analysis, following Braun and Clarke’s six-phase approach (25). This method balances analytic flexibility with rigor, making it well-suited for identifying complex patterns that may not be immediately visible, particularly when exploring nuanced barriers and facilitators across diverse implementation environments (26, 27). This approach enabled the coding team to iteratively develop and refine themes grounded in participant accounts, supporting an analysis that was responsive to local variation and sensitive to broader cross-site dynamics. Two researchers (WB and SES) independently familiarized themselves with the data by reviewing and verifying the accuracy of interview transcripts, which were uploaded into Atlas.ti to facilitate collaborative coding. Coders initially double-coded one transcript, then met to align codes, reconcile coding differences, and define a shared approach. Each coder subsequently analyzed a subset of transcripts independently, reconvening regularly to review coding decisions and refine the code structure (Multimedia Appendix 1). To enhance transparency and illustrate how participant voices informed theme development, representative quotes are provided in Multimedia Appendix 2.

Themes and sub-themes were developed inductively through discussion and initial versions were visualized using a thematic map created in Miro (Multimedia Appendix 3). Subthemes were then organized by whether the underlying codes appeared at timepoint 1 only (interview 1), timepoint 2 only (interview 2), or at both timepoints. This allowed for temporal analysis of subthemes to explore how overarching themes within the project changed over the project. Theme refinement followed Braun and Clarke’s five-question recursive review (28), which included refining names of themes and subthemes, elevating or demoting themes and/or subthemes, and combining themes and subthemes if researchers believed they addressed similar underlying constructs.

### Survey data

Quantitative data were analyzed using descriptive statistics, including the frequency and percentage of respondents endorsing specific options (e.g., “Yes”, “Meaningful,” “Very Meaningful,” or “Agree”). Likert-type items were collapsed where appropriate to improve interpretability (e.g., combining “Meaningful,” “Moderately Meaningful,” and “Very Meaningful”). Survey data were used to identify cross-site patterns and site-specific changes over time. Quantitative data were not intended for inferential testing but provided descriptive context to complement qualitative themes and describe changes in salience for subthemes over time. Although all sites responded to the same questions in the interview, the interview format relies on participants to volunteer information, meaning they may choose to focus on different barriers/and or facilitators. The survey data allows for more comparisons across sites.

### Mixed-methods matrix display

To integrate qualitative and quantitative findings, we developed a matrix aligning qualitative data from interviews with quantitative data from surveys. Subthemes were coded as facilitators (+), barriers (−), mixed (+/−) or neutral depending on their context within themes. Survey questions that aligned with the themes and subthemes from qualitative analysis were determined by researcher consensus (WB and SES). Some survey questions did not overlap with interview data, and those questions were excluded from the mixed-methods analysis.

## Results

### Overview

Integration of survey and interview data identified eight overall themes that are described below. Qualitative interviews provided detailed accounts of implementation experiences, while survey data quantified the prevalence and temporal patterns of these themes and subthemes across sites. The survey findings enabled tracking of changes over time, such as the decline in cross-site collaboration or the persistence of workforce challenges affecting most sites over the study period. These quantitative patterns presented in Table 2 are integrated within the thematic results and demonstrated how implementation dynamics shifted across project phases.

### Vendors supplemented sites’ work

Vendors were contracted by sites to supplement expertise and staffing capacity that were not available internally. They provided technical knowledge, implementation support, and staff to move projects forward, particularly during early phases. In this respect, vendors were viewed as an important resource for filling gaps that sites could not address on their own. At the same time, some technology vendors were often unwilling or unable to customize products, which led to challenges in these products addressing local needs. For example, many vendors would or could not adapt tools for sites’ communities. Many sites requested user-level data collected by technology vendors; but data that was rarely provided. Respondents emphasized that access to more data and granular data from the DHMIs could have informed local decision-making, for example by identifying usage patterns, monitoring engagement over time, and tailoring outreach to underserved groups. The absence of such data limited sites’ ability to adapt technologies to their populations and to evaluate whether implementation strategies were effective. Workflow and communication mismatches also emerged where early in the project vendors moved faster than site approval processes, creating project delays and frustration among sites. By the end of the project, the challenge had shifted: communication between vendors and sites had slowed, and some project leads noted difficulty receiving timely responses. Despite communication tensions, sites reported fewer difficulties with vendors and increased vendor success by the final stages of their projects in both interviews and surveys.

### Sustaining a workforce proved challenging

Workforce challenges, such as shortages and bandwidth, intensified during and immediately after the COVID-19 pandemic but remained persistent throughout the initiative. Dedicated staffing, defined as personnel whose primary role was supporting the Help@Hand project, were considered integral to success across all stages. Surveys underscored the persistence of these challenges, with workforce shortages reported by nearly all sites in the early waves and although reduced somewhat, was still consistently high across all timepoints. Sites consistently reported struggles when staff were spread across multiple projects and responsibilities. High turnover within sites, particularly difficult during and immediately after the COVID-19 pandemic, led to a persistent “knowledge drain”, as experienced staff left and were difficult to replace, with some positions remaining vacant for long periods. Onboarding and training new team members, many of whom were simultaneously learning new digital and implementation skills on the job, placed additional strain on remaining staff. Although staffing pressures became somewhat less salient later in the project, survey data showed that three-quarters of sites still reported limited workforce capacity in the final year, and many sites remained understaffed for the duration of the initiative.

### Sites operated in tight funding environments

Sites described feeling constrained by the funding available to them across the project and used resources and funds from other projects (Medi-Cal Realignment, other mental health initiatives) to supplement their activities for sustainment. Stakeholders, including community members and county, city, and state government leadership, were critical of the program and how money was being spent, and sites often felt scrutinized. In the closeout phase of the project, sites sought funding to sustain their projects; however, considerable uncertainty around the future of innovation funding in California limited opportunities for digital health. By the closeout phase, funding constraints had become particularly acute with sites describing trade-offs between extending licenses for DMHIs, investing in marketing, or hiring staff and consultants. Survey data confirmed this tightening over time, as reports of new or external funding became increasingly rare across successive waves.

### Community outreach and marketing required new and multiple approaches

Across the final three time points, most sites reported that they outreached to organizations, partner groups or members of the community who might be expected to benefit from the Help@Hand project. Successful outreach also meant collaborating with community organizations that serviced populations of interest. In-person outreach, which included tabling at community events, live demonstrations, and one-on-one support, was viewed as effective. Without in-person engagement, flyers and brochures alone were viewed as insufficient. Successful outreach required understanding the needs of the community and their digital access. Conducting needs assessments were a useful facilitator to gain this knowledge. Teams described adjusting formats, locations, and timing of events (e.g., evenings/weekends) and adapting materials to meet language and literacy needs of their local communities. By the midpoint of the Help@Hand project, sites noted taking a multimodal approach to community outreach supported equity and inclusion. Overall, few sites reported challenges in marketing, particularly at the later stages of the project. Sites preferred media platforms like social media posts or Google ads over radio or physical advertising, especially as these types of new media allowed targeting or focus on specific communities. Distributing devices or offering hands-on technology support was a useful form of outreach, especially to create trust and relationships for further engagement.

### Project management had a central role in the project

Respondents reported that the centralized project management entity provided support in navigating complex contracting processes and coordination with external vendors, particularly during early implementation. This was especially valuable for sites with limited experience in digital procurement. However, at the earlier stages of the project, project management’s centrality created difficulties for sites by adding an extra layer of bureaucracy including data sharing agreements and compliance regulations. Surveys reflected this ambivalence across the project timeline with respondents identifying project management as both a source of coordination and, at times, of delays. Communication challenges related to budgets and documentation were also reported across the life of the project. In contrast, project management was useful for coordinating meetings and tasks within sites, such as ensuring deadlines were met for reports.

### Collaborations were both supported and disrupted by organizational factors

Collaboration occurred across the Help@Hand project and within sites but showed a marked decline as the project progressed. Half of all sites reported collaborations with other sites at Survey 2; however, this dropped to approximately a quarter of sites by the end of the project. Within collaborations, pooling resources with other sites such as sharing unused app licenses or repurposing materials developed by other sites, was considered extremely useful. Pooling knowledge between sites was also viewed as critical, which included using the experiences of other project leads and managers, or replicating strategies other had implemented successfully. Sites also reported a lack of opportunities to reflect on the outcomes from the work that other sites had undertaken and felt that there could have been more chances to collaborate. This was reflected in reports from some sites at later stages of the project that wished that they had been able to utilize resources from other sites to solve their own local problems. Similarly, at later stages sites described synchronizing processes, such as meetings or communication channels, was difficult across sites. Intra-site collaboration was also critical, with cross-departmental collaboration supporting important project activities such as outreach and resource sharing. However, unresponsiveness and indecisiveness from site leadership and poor cross-departmental communication created challenges for implementation and this was understood to reflect a lack of cross-site buy-in.

### Digital equity was crucial for the community

Digital equity was a persistent theme, emerging as both a barrier and a vehicle for community support. At the intervention-level, many tools were not designed with linguistic or cultural inclusiveness in mind, and few supported offline use or integrations with clinical systems. This mismatch between off-the-shelf technology and community realities restricted a sites’ ability to engage marginalized residents, particularly in communities with limited broadband access, deaf or hard of hearing individuals and other marginalized groups. This was exacerbated by the lack of tailoring that vendors were willing or able to deliver to sites, despite efforts made by sites to understand needs and preferences of communities. However, reports from sites reflected variation in vendor responsiveness, with some describing vendors as flexible and collaborative partners in supporting implementation.

Structurally, project leads described access to devices, internet connectivity, and digital literacy as barriers. Many sites introduced device lending or distribution to close access gaps, though this introduced procurement, ownership, and logistics challenges. In parallel, digital literacy efforts grew over time. At earlier stages of the project, a third of all sites were providing digital literacy trainings; however, this increased to approximately half of all sites by later stages. Digital literacy was necessary, as a lack of digital literacy was considered a significant barrier to community outreach for many sites. While digital literacy training was important for providing access to the Help@Hand project, sites also reported that this supported their community members beyond Help@Hand, such as in navigating sites’ online portals or creating resumes for job applications.

## Discussion

Implementing DMHIs is a challenging and complex process, especially with public systems. This study contributes to the field of implementation science by presenting mixed-methods implementation outcome data from the California-wide Help@Hand DMHI initiative. We report on barriers to implementation within and across sites, noting how these barriers shifted as projects moved from preparation to implementation to sustainability planning. The evolution of the project over time reflected local circumstances, community needs, available infrastructure, and priorities. This evolution reflected an interpretive process that influenced project goals, tool selection, target audiences, and implementation approaches, aligning with implementation literature that highlights implementation as a meaning-making process embedded in context (22). Our findings suggest that DMHI implementation is not a linear process of technology adoption, but an interpretive, constructive, and adaptive endeavor. We conclude by reflecting on these dynamics to inform future large-scale implementation efforts and the broader field of implementation science.

External vendors were a critical support for sites across the life of the Help@Hand project. They provided expertise to rapidly develop and deliver DMHIs where sites lacked in-house capacity or knowledge. At the same time, technology vendor’s responsiveness, willingness to customize, and control over data access shaped what could be implemented, to whom, and what could be learned. Differences in the pace of work between vendors and site staff created ongoing challenges. Some respondents reported that vendors struggled with the slow pace of approvals within their departments, which was described as the norm for ‘government work’. Misalignment in workflows and work practices is a common occurrence in digital health (29), which can cause delays to implementation and exacerbate tensions between parties. Conversely, while this misalignment may have been a barrier during the early implementation phase of the Help@Hand project, the autonomy for vendors to update and support the DMHI independently could be a demonstrable advantage in other contexts (30). Team effectiveness research may support the development of shared mental models within teams i.e., developing a common understanding of the problem (31), thereby improving communication between parties and clarifying the tempo of work. This can be achieved through various team training strategies (32, 33) and would allow each party the opportunity to set priorities, goals, and preferences for how they want to successfully implement the DMHI.

The reliance on vendors was intensified by another persistent challenge: limited staffing and workforce turnover, which eroded the internal capacity sites needed to manage and sustain implementation on their own (34). Sustaining the workforce during and immediately post-COVID pandemic was a significant challenge for all involved sites. While this dynamic shifted over the course of the project and became less salient over time, three quarters of sites still reported that they operated with limited staffing in the final year of the project. Staff turnover was a primary driver of this challenge, which resulted in a knowledge drain through the loss of experienced staff who were difficult to replace, so much so that many sites remained understaffed for the entire Help@Hand project. The loss of experienced staff reduced organizational capacity by reducing the number of individuals who had the skills and local knowledge required to implement a DMHI in the public system. This also has implications on funding stability and relational connections, both factors that are built-up over long periods of time and vulnerable to short term disruptions (35). This aligns with prior work in public schools (36) and community-based organizations (37), which also demonstrated that staff turnover can lead to the the loss of local knowledge and the professional relationships needed to implement interventions within a community.

Dedicated staffing was perceived as a core factor for success within sites. Despite this, many staff reported that they had competing priorities that prevented this, which is a common challenge to sustainability for implementation projects in public health and community-based systems (38–41). Turnover exacerbated this problem, as Help@Hand were frequently required to instead invest time into onboarding and training new staff as replacements. Furthermore, contributing to tasks outside of the Help@Hand project took away the specialty digital expertise gained and necessary for DMHI implementation. Additionally, when these staff left the project, it took that expertise away altogether. While turnover is a known concern in any organization, its impact was disproportionately large in smaller teams requiring specialized knowledge to succeed. This instability intersected with workforce composition, as previous analysis of the Help@Hand project found that Peers hired for lived experience were at risk of relapse and turnover without adequate structural support (34).

Turnover also has a financial impact due to the resources required to hire and train new staff (35, 38). The loss of organizational capacity furthered the need for sites to work with vendors to supplement their knowledge and workforce, even if they may have preferred to keep some tasks internal. Building organizational capacity would give sites more opportunity to undertake implementation activities internally and reduce reliance on vendors who were not consistently flexible.

Organizational capacity was not only a matter of staff skills and stability, but also of financial resources. Sites operated under funding constraints that shaped how much they could invest in staffing, technology, and outreach. This resulted in sites making trade-offs about where to allocate funding; for example some sites had to choose between extending licenses for DMHIs, marketing, or hiring staff and consultants. Sites were especially cognizant of these funding constraints due to them operating in a public health system where stakeholders, often scrutinized the premise and costs of the Help@Hand project. Similar patterns have been observed when implementing DMHIs within the National Health Service in the UK, where project leads expressed that leadership’s expectations and criticisms grew proportionally to the size of the funding (42). In this project, sites were able to use other funding sources, drawing on Medicaid, Medi-Cal or other county funds. While this was necessary in the short-term, it was not sustainable in the long-term. In this study, just over one quarter of sites reported having secured additional funding external to the Help@Hand project to support their initiatives at the midpoint of this study; however, none had applied for funding or been successful in acquiring funding at the end of the project. Sustainability is critical for DMHIs where projects commonly stall in the early implementation phases, termed ‘pilotitis’ (43), and fail to be integrated into the existing health system and scale-up appropriately. Prior implementation work in community-based organizations has demonstrated the importance to sustainability of equipping providers with the skills to identify potential funding sources and training them to effectively apply for them (44). Supporting sites through developing funding capacity may have alleviated some of the anxiety around sustainability and given project managers the confidence to identify and apply-to relevant innovation funding for DMHI-related work.

Funding challenges were intertwined with the governance structure of the project where a centralized coordinating body sought to manage resources and oversight while local sites attempted to adapt initiatives to their own contexts. The Help@Hand project was governed through a hybrid model: centrally-funded through a coordinating agency, but locally executed through individual county and city healthcare agencies. Sites were tasked with realizing an ambitious vision for digital mental health within the context of their own infrastructure and capacity. A persistent tension emerged between the initiative’s broader goals such as system transformation and statewide innovation, and the realities of what sites could feasibly achieve given their own staff, vendor support, and institutional authority. This is a common challenge for multilevel implementation projects where outer context priorities do not align with inner context readiness and capacity (45). This can have a constraining effect on projects where the actual financial and governance model underpinning a project can unintentionally constrain local adaptation and create barriers to implementation (46). While the centralized coordinating body offered contracting support and facilitated recurring meetings, supports were often described as bureaucratic rather than enabling, adding steps to already complex processes without alleviating local burdens. Sites frequently operated independently, improvising their own solutions and developing their own materials, workflows, and protocols in the absence of centralized templates or implementation tools. In parallel, sites also overcame shared challenges by pooling resources, such as sharing unused product licenses. Similar to hybrid models in other contexts (47, 48), increased bureaucracy introduced by centralized coordination caused delays and frustration. These dynamics highlight that hybrid governance models require not only coordination structures but also strong local leadership buy-in, without which cross-organizational collaboration and knowledge sharing may falter. Leadership has a significant role in fostering a positive implementation climate (49), particularly in aligning goals and expectations across levels and between agencies (45). Ultimately, our findings suggest that for large, multi-agency projects over a large geographic area such as California, centralized governance on its own is insufficient to support system transformation and DMHI implementation. Centralized coordination must be accompanied by flexible, localized pathways to action that support bottom-up innovation and adaptation in diverse settings (10). At the same time, centralized coordination may be more effective for some functions than others, particularly when it complements rather than duplicates local efforts. The effectiveness of centralized governance appears contingent on the quality of the coordination itself and its ability to fill gaps, streamline processes, and avoid adding redundant layers to already complex systems.

Digital equity provided a critical lens through which sites enacted and redefined their activities. Equity was one of the most frequently cited project goals across sites, particularly with respect to geographic, linguistic, and economic access. How sites operationalized this goal varied depending on their capacity, partnerships, and understanding of community needs. Some pursued robust structural efforts to reduce access barriers at scale, such as hotspot lending libraries, large-scale device distribution programs, and multilingual outreach campaigns. Others engaged in more constrained activities, including distributing flyers in a few community settings or conducting brief outreach to underserved populations. Across the project, sites demonstrated a genuine commitment to equity, but their efforts often lacked the resources necessary to reach or sustain engagement with marginalized populations. These patterns align with findings from other DMHI initiatives, where addressing equity has been addressed by lowering structural barriers to access. Public libraries, for example, have been leveraged as trusted sites for adolescent engagement (49, 50). For youth experiencing homelessness, distributing phones and providing mobile service has expanded both connectivity and access to digital interventions (52, 53). Digital literacy training has likewise been shown to improve both uptake and mental health outcomes (54).

Within the Help@Hand project, sites recognized the importance of multimodal outreach and increasingly found that targeted strategies, such as social media or Google ads, were especially effective for engaging overlooked communities. Our data extends the literature by demonstrating that multimodal and targeted approaches can reach linguistically and culturally diverse groups and geographically distinct and marginalized populations (55–57). Still, outreach strategies alone could not overcome limitations embedded in the DMHIs themselves. Many tools were not designed with linguistic or cultural inclusiveness, accessibility, or offline functionality in mind, constraining how far equity goals could be realized in practice. This mismatch between off-the-shelf technology and community realities restricted sites’ ability to engage marginalized residents, particularly in communities with limited broadband access, deaf or hard of hearing individuals and other marginalized groups. These challenges reflect a broader pattern in the field: DMHIs are often not ready or available to meet the needs of diverse populations. Prior research has identified persistent gaps in Spanish-language mental health apps (58) and highlighted the limited adaptation of tools for marginalized and underserved groups (59). While sites were motivated to close equity gaps, some lacked the partnerships or capacity to reach disparate groups. In these cases, equity remained an aspirational aim, rather than an actionable strategy. As others have noted, DMHIs do not guarantee equity. In some cases, it may entrench existing disparities or create new forms of exclusion if not paired with intentional capacity-building (60). The site differences in Help@Hand underscore that equity was not an inherent property of DMHIs, but something actively constructed through implementation.

Altogether, the themes presented herein suggest that DMHI implementation cannot be understood as a linear process of adopting ready-made tools. Instead, implementation emerges through ongoing interpretation and configurational work, in which vendors, staff, funding, governance, and equity considerations continuously shape one another. This interpretive work—including defining target populations, selecting tools, and operationalizing equity goals—shaped every aspect of implementation. This pattern aligns with conceptualizations of health technologies as socio-technical assemblages: not discrete objects with fixed properties, but dynamic entities that take shape through interactions between human and non-human actors (22, 61–63). In this view, the “same” technology becomes fundamentally different when enacted in different contexts through ongoing negotiation of meaning, scope, and boundaries. Our findings extend this insight to implementation itself, which can be thought of as a socio-technical assemblage produced through the interplay of sites, vendors, policies, infrastructure, and community contexts. Infrastructure, in particular, was not background but constitutive: staffing, contracting, integration, and access to analytics determined what the intervention could be. Sites did not simply succeed or fail at implementation; they engaged in continuous adaptation, modification, and sense-making that reshaped both the technology and their organizational practices. These insights resonate with established implementation science frameworks, including the Exploration, Preparation, Implementation, and Sustainment (EPIS) framework (64), the Consolidated Framework for Implementation Research (CFIR) (24), the Dynamic Sustainability Framework (65), and the Nonadoption, Abandonment, Scale-Up, Spread, and Sustainability (NASSS) framework (22). Each emphasizes that implementation is contingent, multilevel, and evolving rather than predetermined. Future DMHI initiatives should therefore recognize and support interpretive and configurational work, rather than assuming that standardized tools or implementation models can be uniformly deployed across diverse settings.

### Limitations

While the study provides insights into the implementation of DMHIs, several limitations should be noted. The findings are based on self-reported data from project leads, which may be subject to bias, including recall bias, social desirability. The reliance on a single primary informant per site may limit the comprehensiveness of perspective. A broader range of stakeholders, such as frontline staff, end users, and external partners, could provide a more comprehensive and nuanced understanding of implementation dynamics. Using the project lead as the sole informant may also privilege managerial perspectives over others The timing of data collection meant that data analysis reflected the latter stages of the project, and the pre-implementation/preparation stages of Help@Hand are not as comprehensively covered. Finally, California’s public behavioral health system operates under specific funding models, governance structures, and policy environments. These conditions may differ significantly from those in other states, private healthcare systems, or international settings where mental health service funding and delivery is different.

## Conclusion

This longitudinal evaluation of California’s Help@Hand initiative provides insights into the implementation of DMHIs within public behavioral health systems. Our findings demonstrate that barriers to and facilitators of implementation can be temporal and specific to a local context. Implementation was a process of socio-technical co-construction shaped by multiple, interrelated factors. Successful implementation depended less on the properties of digital tools themselves and more on the capacity of systems to sustain expertise, align funding and governance, create opportunities for learning, and embed equity into practice.

## Supplementary Material

This is a list of supplementary files associated with this preprint. Click to download.

- HelpAtHandCrossCountyEvaluationMultimediaAppendix.xlsx
- HelpAtHandCrossCountyEvaluationMultimediaAppendix2.docx
- HelpAtHandCrossCountyEvaluationMultimediaAppendix3.docx
- SRQRChecklist.docx

## Figures and Tables

**Table 1:** Data collection timeline by activity and respondents.

Eval Type	Administered	Reporting Period	# of Respondents
Interview 1	April – May 2022	Since April 2021	10
Survey 1	July – August 2022	Since July 2021	12
Survey 2	Oct – Dec 2022	Since Jan. 2022	12
Survey 3	March – April 2023	Since Jan. 2023	11
Interview 2	June – July 2023	Since June 2022	11
Survey 4	Oct – Nov 2023	Since Apr. 2023	8

Note: Some sites concluded their participation in Help@Hand before the end of the overall project period, leading to fewer responses over time.

**Table 2:** Joint Display table combining interview and survey data across timepoints.

Theme	Interview 1: 2022	Interview 2: 2023	Survey 1 (mid 2022)	Survey 2 (late 2022)	Survey 3 (early 2023)	Survey 4 (late 2023)
**Vendors supplemented sites’ work.**	**+ Subtheme**: Vendors were easy and flexible to work with. **+ Subtheme**: Contracting streamlined sites’ work by utilizing external expertise not present. **- Subtheme**: Vendors displayed a lack of flexibility on customization. **- Subtheme**: Sites desired more data from the apps.		+ Successfully executed a contract with a vendor: 10/12 sites Contracting for products, like those included in Help@Hand, required knowledge that had not been present in current teams: 8/12 agreed - Experienced contracting difficulties: 7/12 sites	+ Successfully executed a contract with a vendor: 7/12 sites Contracting for products, like those included in Help@Hand, required knowledge that had not been present in current teams: 8/12 found this meaningful/very meaningful - Experienced contracting difficulties: 7/12 sites	+ Successfully executed a contract with a vendor: 4/11 sites Contracting for products, like those included in Help@Hand, required knowledge that had not been present in current teams: 6/11 found this meaningful/very meaningful - Experienced contracting difficulties: 3/11 sites	+ Successfully executed a contract with a vendor: 6/8 sites Contracting for products, like those included in Help@Hand, required knowledge that had not been present in current teams: 4/8 found this meaningful/very meaningful - Experienced contracting difficulties: 4/8 sites
	**- Subtheme**: There was a difference between sites and vendors in speed of work.	**- Subtheme**: Communication could be difficult between sites and vendors.				
**Sustaining a workforce proved challenging.**	**- Subtheme**: Sites experienced lack of dedicated staffing, resulting in competing priorities and time constraints. **- Subtheme**: Turnover was common, and sites remained understaffed. **- Subtheme**: Onboarding new staff was intensive. **- Subtheme**: Losing experienced/expert staff caused a knowledge drain.		Dedicated staffing is necessary for project success: 7/12 sites agreed - Experienced staff shortages: 12/12 sites	Dedicated staffing is necessary for project success: 12/12 sites felt this was meaningful or very meaningful - Experienced staff shortages: 12/12 sites	Dedicated staffing is necessary for project success: 10/11 sites felt this was meaningful or very meaningful - Experienced staff shortages: 5/11 sites	Dedicated staffing is necessary for project success: 8/8 sites felt this was meaningful or very meaningful - Experienced staff shortages: 6/8 sites
**Sites operated in tight funding environments.**		**Subtheme**: Sites were actively seeking funding for project continuation. **- Subtheme**: Sites experienced uncertainty around future innovation funding.	Obtained additional funding to support sustainability of activities developed through Help@Hand: 4/12 sites	Obtained additional funding to support sustainability of activities developed through Help@Hand: 1/12 sites	Obtained additional funding to support sustainability of activities developed through Help@Hand: 1/11 sites Submitted a grant proposal to obtain non-MHSA funding to support Help@Hand activities: 0/11 sites	Obtained additional funding to support sustainability of activities developed through Help@Hand: 0/8 sites Submitted a grant proposal to obtain non-MHSA funding to support Help@Hand activities: 0/8 sites
	**+ Subtheme**: Sites leveraged other resources to fill gaps. **- Subtheme**: Limited funding was available. **- Subtheme**: Stakeholders scrutinized use of funding.					
**Community outreach and marketing required new and multiple approaches.**	**+ Subtheme**: In-person outreach built strong relationships. **+ Subtheme**: Outreach involved multiple collaborations. **+ Subtheme**: Digital equity supported outreach. **+ Subtheme**: Outreach involved assessing and meeting community needs. **- Subtheme**: Sites experienced uncertainty around traditional marketing approaches.			+ Conducted outreach to organizations or partner groups about the Help@Hand program: 7/12 sites + Conducted outreach to members of the community who might be expected to benefit from the Help@Hand technology: 9/12 Sites - Experienced challenges related to outreach: 3/12 sites	+ Conducted outreach to organizations or partner groups about the Help@Hand program: 7/11 sites + Conducted outreach to members of the community who might be expected to benefit from the Help@Hand technology: 8/11 sites - Experienced challenges related to outreach: 4/11 sites	+ Conducted outreach to organizations or partner groups about the Help@Hand program: 4/8 sites + Conducted outreach to members of the community who might be expected to benefit from the Help@Hand technology: 6/8 sites - Experienced challenges related to outreach: 2/8 sites
		**+ Subtheme**: Targeted advertising was useful at reaching specific audiences.		- Experienced challenges related to marketing: 4/12 sites	- Experienced challenges related to marketing: 1/11 sites	- Experienced challenges related to marketing: 3/8 sites
**Project management had a central role in the project.**	**- Subtheme**: Additional layers of decision-making created complexity.					
	**+ Subtheme**: The project manager supported the coordination of internal sites’ work. **- Subtheme**: Communication difficulties created delays.					
**Collaborations were both supported and disrupted by organizational factors.**	**+ Subtheme**: Pooling knowledge was beneficial. **+ Subtheme**: Pooling resources helped to overcome shared challenges. **- Subtheme**: Sites experienced limited opportunities to reflect on other site processes and results. **- Subtheme**: Departmental leadership lack-of-buy-in, unresponsiveness or indecisiveness caused delays.		+ Had cross-site collaboration to support Help@Hand activities: 6/12 sites agreed	+ Had cross-site collaboration to support Help@Hand activities: 3/11 sites agreed	+ Had cross-site collaboration to support Help@Hand activities: 2/8 of sites agreed	
		**+ Subtheme**: Collaborating across departments supported outreach. **- Subtheme**: Sites experienced missed opportunities for cross-support. **- Subtheme**: Synchronizing sites’ processes was difficult.				
**Digital equity was crucial for the community.**	**+ Subtheme**: Digital literacy training helped individuals beyond the Help@Hand project.					
	**- Subtheme**: The digital divide was a barrier to reaching the community.	+ Provided digital literacy training to the community: 4/12 sites	+ Provided digital literacy training to the community: 6/12 sites	+ Provided digital literacy training to the community: 5/11 sites	+ Provided digital literacy training to the community: 6/8 sites	

Note. Themes, subthemes, and survey questions were coded as facilitators (+), barriers (−), mixed (+/−) or neutral

## Data Availability

Anonymized and deidentified quantitative material may be made available upon reasonable request. Access to raw qualitative data may not be possible due to the potential for participants to be revealed related to responses within text.
